# Supplementary material for: AffyMAPSDetector: a software tool to characterize Affymetrix GeneChip™ expression arrays with respect to SNPs
Source: BMC Bioinformatics. 2007 Jul 30;8:276. doi: 10.1186/1471-2105-8-276 (PMC1959249; doi:10.1186/1471-2105-8-276)
Supplement: Additional file 13 — AffyMAPSDetector v1 source code. AffyMAPSDetector source code. [file 1471-2105-8-276-S13.zip › AffyMAPSDetector_v1_src/doc/affychip/snpdetector/model/event/class-use/StatusEvent.html]

Uses of Class affychip.snpdetector.model.event.StatusEvent


|  |  |  |  |
| --- | --- | --- | --- |
| **Overview** \| **Package** \| **Class** \| **Use** \| **Tree** \| **Deprecated** \| **Index** \| **Help** | | |  |
| PREV   NEXT | **FRAMES**    **NO FRAMES**     **All Classes** |  |  |


---


## **Uses of Class affychip.snpdetector.model.event.StatusEvent**

|  |  |
| --- | --- |
| Packages that use StatusEvent | |
| **affychip.snpdetector.model** |  |
| **affychip.snpdetector.model.event** |  |
| **affychip.snpdetector.ui** |  |

Uses of StatusEvent in affychip.snpdetector.model

|  |  |
| --- | --- |
| Methods in affychip.snpdetector.model with parameters of type StatusEvent | |
| `void` | `SnpMatcher.fireStatusUpdateEvent(StatusEvent e)` |

Uses of StatusEvent in affychip.snpdetector.model.event

|  |  |
| --- | --- |
| Methods in affychip.snpdetector.model.event with parameters of type StatusEvent | |
| `void` | `StatusEventListener.statusUpdate(StatusEvent pe)` |

Uses of StatusEvent in affychip.snpdetector.ui

|  |  |
| --- | --- |
| Methods in affychip.snpdetector.ui with parameters of type StatusEvent | |
| `void` | `AffyMapsDetectorFrame.statusUpdate(StatusEvent e)` |

---


|  |  |  |  |
| --- | --- | --- | --- |
| **Overview** \| **Package** \| **Class** \| **Use** \| **Tree** \| **Deprecated** \| **Index** \| **Help** | | |  |
| PREV   NEXT | **FRAMES**    **NO FRAMES**     **All Classes** |  |  |


---
